# Biological phase separation: cell biology meets biophysics

**DOI:** 10.1007/s12551-020-00680-x

**Published:** 2020-03-18

**Authors:** Takuya Yoshizawa, Ryu-Suke Nozawa, Tony Z. Jia, Tomohide Saio, Eiichiro Mori

**Affiliations:** 1 Department of Biotechnology, College of Life Sciences, Ritsumeikan University, Kusatsu, Shiga Japan; 2 Division of Experimental Pathology, Cancer Institute of the Japanese Foundation for Cancer Research (JFCR), Tokyo, Japan; 3 Earth-Life Science Institute, Tokyo Institute of Technology, Tokyo, Japan; 4 Blue Marble Space Institute of Science, Seattle, WA USA; 5 Department of Chemistry, Faculty of Science, Hokkaido University, Sapporo, Hokkaido Japan; 6 Department of Future Basic Medicine, Nara Medical University, Kashihara, Nara Japan

**Keywords:** Liquid-liquid phase separation (LLPS), Membrane-less organelle, Low-complexity (LC) domain, Intrinsically disordered region/protein (IDR/IDP)

## Abstract

Progress in development of biophysical analytic approaches has recently crossed paths with macromolecule condensates in cells. These cell condensates, typically termed liquid-like droplets, are formed by liquid-liquid phase separation (LLPS). More and more cell biologists now recognize that many of the membrane-less organelles observed in cells are formed by LLPS caused by interactions between proteins and nucleic acids. However, the detailed biophysical processes within the cell that lead to these assemblies remain largely unexplored. In this review, we evaluate recent discoveries related to biological phase separation including stress granule formation, chromatin regulation, and processes in the origin and evolution of life. We also discuss the potential issues and technical advancements required to properly study biological phase separation.

## The formation and regulation of membrane-less cellular organelles

In eukaryotic evolution, cells gained a lipid bilayer nuclear membrane, separating the nucleus from the cytoplasm. Such a nuclear membrane enables segregation of certain factors (including proteins and metabolites) in their proper locations and provides selective trafficking between the nucleus and the cytoplasm. Although it may seem that the presence of a physical boundary, such as a nuclear membrane, is the only way compartments can be formed and provide trafficking control within cells, cells in fact have another option: liquid-liquid phase separation (LLPS). Indeed, a large number of organelles including nucleoli, Cajal bodies, promyelocytic leukemia (PML) bodies, processing bodies (P-bodies), and stress granules are membrane-less. It has become clear recently that these organelles are formed through LLPS (Banani et al. 2017; Shin and Brangwynne 2017). LLPS defines distinct compartments to efficiently organize cellular processes by concentrating certain factors in their proper place without interfering with one another in the complex and heterogeneous environment within a cell. These cellular bodies are dissolved during mitosis and reformed in the next round of the cell cycle (Rai et al. 2018). They are also reversible, unlike aggregates, and appear to be in a viscoelastic-dynamic fluid state, which gives them plasticity and flexibility.

Most LLPS researches are focused on events in eukaryotic cells. Even in prokaryotic cells, LLPS system may be utilized for cell division (Monterroso et al. 2016, 2019). There should be a number of undiscovered LLPS systems in both eukaryotes and prokaryotes (Alberti 2017a). As biological LLPS systems and organelles are incredibly complex and often involve a suite of co-interacting proteins, molecules, and other co-factors, we refer to the relevant interacting molecules and some of the associated processes by their commonly used abbreviations henceforth in this text. Each abbreviation is defined at first mention in the text. They are also compiled in alphabetical order by sections within this manuscript. This list, with relevant page number information, is included in Appendix Table 1 and provides readers with a quick way to reference the relevant abbreviations when a specific process is described in the text.

﻿The formation of cellular membrane-less compartments is driven by multivalent interactions among nucleotides or amino acids (Banani et al. 2017; Shin and Brangwynne 2017). A disordered region of proteins termed the intrinsically disordered region (IDR), or the low-complexity (LC) domain, facilitates assembly (Kato et al. 2012; Forman-Kay and Mittag 2013; Nott et al. 2015). RNA also serves as a seed in defining the location of the phase-separated compartment. For example, the largest nuclear structure for ribosome biogenesis, the nucleolus, is formed near ribosomal RNA (rRNA) transcription sites. When rRNAs are artificially transcribed elsewhere in the chromosome, a new nucleolus-like condensate is formed at that site (Karpen et al. 1988; Oakes et al. 2006), while nucleolar component assemblies at random nuclear positions are observed in inhibiting rRNA transcription or deletion of ribosomal DNA (Berry et al. 2015; Falahati et al. 2016).

In terms of regulatory parameters for LLPS in cells, in addition to variations in the concentration of the major components that drive the LLPS, variations in microenvironments surrounding the condensates such as temperature and ionic strength are conceivable (Nott et al. 2015) as entropy and electrostatic interactions can be affected by these microenvironmental changes. Post-translational modification of proteins is also an important factor driving the assembly or dissociation of such a condensate. In particular, as the phosphate group contains negative charges, phosphorylation of proteins directly impacts the balance of multivalent electrostatic interactions (Aumiller and Keating 2016). It is interesting to note that historically, the relationship between the nucleolus and cancer has been discussed because the number and shape of nucleoli are altered in almost any type of cancer cell. An enlarged and prominent nucleolus is one of the indicators of cancer diagnoses in histopathology (Montanaro et al. 2008; Sakamoto et al. 2018). Such a phenomenon might indicate that a cancer-specific nuclear environment affects or induces the function and assembly/disassembly of condensates inside cancer cells.

## Stress granule assembly, regulation, and diseases

RNA granules, composed of RNA and RNA-binding proteins (RBPs), such as stress granules (SGs), P-bodies, Cajal bodies, and nuclear speckles, play an important role in the cytoplasm and nucleus (Banani et al. 2017). LLPS of the components (proteins and RNA) drives the formation of such RNA granules, and hence, RNA granules are not surrounded by lipid membranes like other membrane bound organelles. This membrane-less feature of LLPS-driven organelles allows for quick assembly and disassembly reflecting various in-cellular conditions. RNA granules provide high-order function in complex biological systems processing or suppressing specific reactions, and unregulated RNA granules have been strongly linked to diseases. In particular, the transformation of SGs into aggregate-like inclusions is considered to be a major cause in the development of fatal neurodegenerative diseases, such as amyotrophic lateral sclerosis (ALS) and frontotemporal dementia (FTD) (Patel et al. 2015; Alberti and Dormann 2019). Proper regulation of aggregate formation is required to maintain cell homeostasis, thus suggesting that chaperones for SGs are key to the prevention of disease-causing transformations.

In order to preserve the function of cells within an organism, these cells must survive various environmental stresses such as high temperature or oxidative stress. Under the stress conditions, SGs are formed in the cytosol in order to suppress translation. There are two reasons cells produce SGs under stress: (1) to save limited resources and energy for essential functions that more directly combat stress; and (2) to avoid increasing defective ribosomal products because of misfolding or premature termination (Patel et al. 2015; Alberti et al. 2017; Boeynaems et al. 2018). The mechanism of SG assembly occurs through self-assembly of the RBPs including G3BP (G3BP stress granule assembly factor 1 or GTPase-activating protein (SH3 domain)-binding protein 1), fused in sarcoma (FUS), and heterogeneous nuclear ribonucleoprotein A1 (hnRNPA1).

**Fig. 1:**
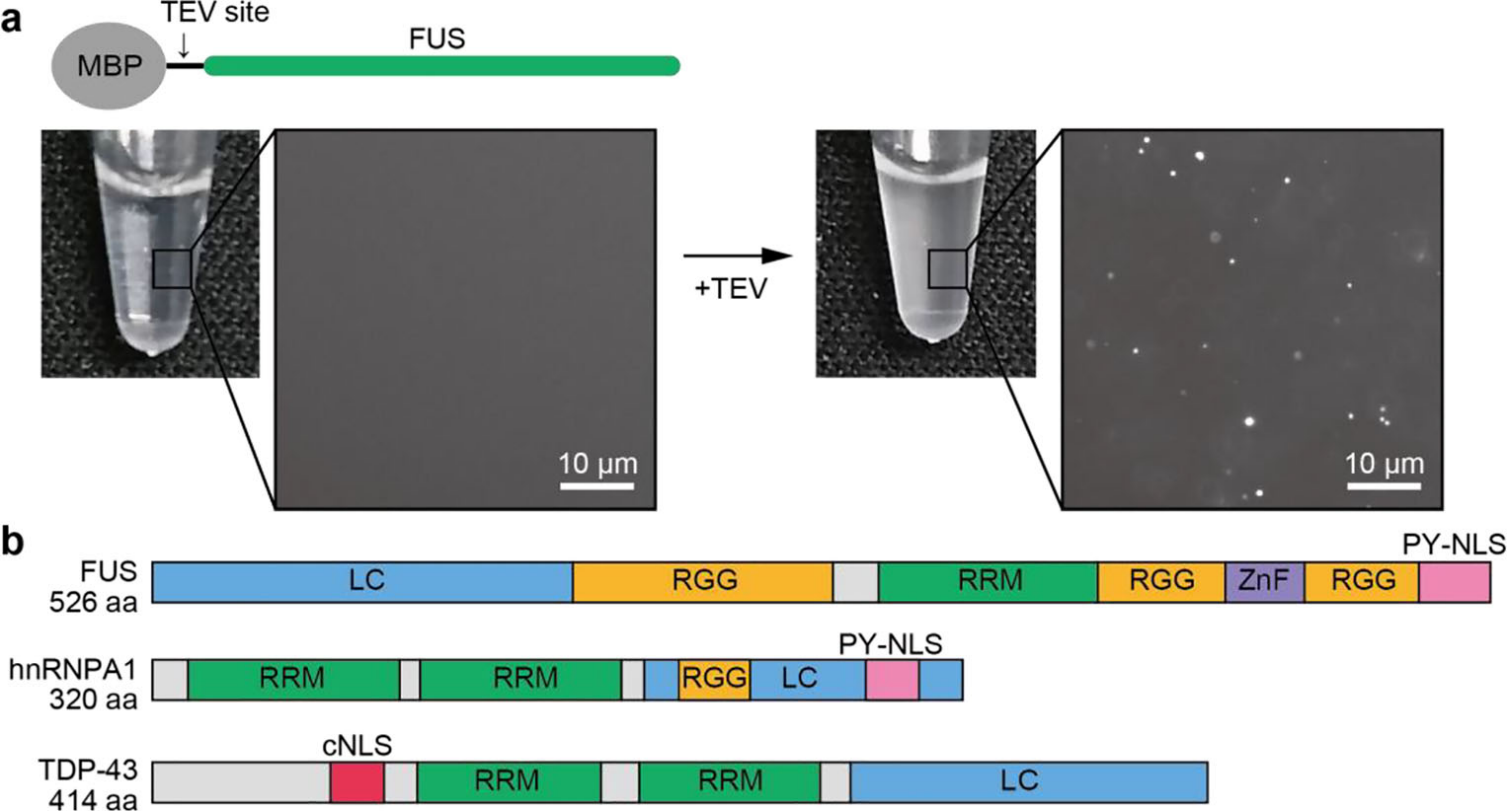
**a** In vitro LLPS initiation of fused in sarcoma (FUS) using maltose-binding protein (MBP)-FUS. A TEV cleavage site was introduced between MBP and FUS (top). MBP-FUS is monodisperse and clear in the test tube. The solution became cloudy after TEV treatment (bottom). Close-up panels show microscope images of the solutions. **b** Domain map of RNA-binding proteins (RBPs) FUS, hnRNPA1, and TDP-43 imported by nuclear import receptors (NIRs). LC, low-complexity domain (blue); RGG, Arginine-Glycine-Glycine repeat (orange); RRM, RNA recognition motif (green); ZnF, zinc finger (purple); PY-NLS, proline-tyrosine nuclear localization signal (pink); cNLS, canonical-NLS (red)

The self-assembly of FUS in relation to the LLPS mechanism has been well-studied. Kato et al. (2012) reported that cross-β polymerization of FUS is essential for the assembly of various RNA granules. This prompted a group of researchers (Yoshizawa et al. 2018; Yoshizawa and Matsumura 2020) to apply a tag-protein system to observe FUS LLPS. In particular, they found that maltose-binding protein (MBP) prevented FUS LLPS as MBP-fused FUS was observed to be soluble and monodisperse. When a TEV protease site was introduced to MBP and FUS, droplet formations, dependent upon increasing free FUS concentration, were observed (Fig. 1a), suggesting that multivalent interactions initiated by the free N-terminus LC domain are the mechanism resulting in the formation of RNA granules. SGs in cells are not only composed of FUS and other RBPs, but also contain mRNA, resulting in a mesh-like network between RBPs and RNAs (Ditlev et al. 2018). Thus, multivalent homo- and hetero-interactions contribute to form SGs. Furthermore, SGs segregate a number of proteins including initiation factors, signaling factors, heat shock proteins, and nuclear import receptors (NIRs) (Zhang et al. 2018). These findings imply that SGs pause multiple cellular systems under stress.

Aberrant SGs have been linked to a variety of fatal diseases. In particular, uncontrolled aggregation of SG proteins is a hallmark of neurodegenerative diseases including ALS and FTD. DNA-binding protein of 43 kDa (TDP-43), an RBP, aggregates were observed in most ALS cases (Harrison and Shorter 2017). Mutations in other RBPs including FUS and hnRNPA1/A2 have been reported in familial neurodegenerative patients. These proteins share an LC domain adjacent to the RNA recognition motif (Fig. 1b). The LC domains are also known as prion-like domains, which natively unfold as monomers when in isolation, but polymerize upon accumulation. Although SGs utilize the self-association force with RBPs to assemble, such assemblies can form harmful amyloid-like polymers under certain conditions, especially in disease patients. Mutations found in ALS patients promote accumulation of RBP or change the physical properties (less reversibility) of SGs (Patel et al. 2015; Mateju et al. 2017). The amount of aberrant SGs increases with age and these SGs can also contain misfolded proteins.

Other than defective SG components, the hexanucleotide repeat expansion (HRE) of chromosome 9 open reading frame 72 (C9orf72) is the most common cause of familial ALS and FTD (Mori et al. 2013). In the C9orf72 HRE, the repeat sequence (GGGGCC)n is translated to dipeptide repeats by repeat-associated non-AUG (RAN) translation. Five types of dipeptides, poly glycine-proline (GP)/proline-alanine (PA)/glycine-arginine (GR)/proline-arginine (PR)/glycine-alanine (GA), are produced from six reading frames in either the sense or the antisense strand. Poly-PR/GR peptides show strong toxicity due to their ability to target and bind to products of LLPS including nucleoli, the nuclear pore complex, and SGs, resulting in their aggregation or disassembly (Freibaum et al. 2015; Lin et al. 2016; Shi et al. 2017). Highly charged and polar poly-GR/PR peptides influence LLPS dynamics. On the other hand, poly-GP/PA has no reported toxicity and poly-GA has shown moderate toxicity involved in p62/ubiquitin-positive inclusions (Freibaum and Taylor 2017).

Due to the toxicity of aberrant SGs, cells require the ability to modulate the disassembly of SGs. Several studies in yeast and fruit fly models revealed that a protein quality control system is utilized to clear SG assembly. SG-recruited ATP-driven chaperone heat shock protein 70 (HSP70) can target misfolded proteins in SGs and lead to the ubiquitin proteasome or autophagy pathway, resulting in aberrant SG disassembly (Alberti et al. 2017). Recent studies have also demonstrated that NIRs play a role as a chaperone for SGs (Guo et al. 2018; Hofweber et al. 2018; Qamar et al. 2018; Yoshizawa et al. 2018). The canonical function of NIR is to transport macromolecules in the cytoplasm into the nucleus. Within the NIR group of proteins, there are twenty proteins in the human karyopherinβ (Kapβ) family with similar molecular weights (ca. 100–130 kDa) and helical topologies. Each Kapβ recognizes a specific nuclear localization signal (NLS) on macromolecules (proteins and RNA) (Soniat and Chook 2015). Importinα/β (Impα/β) and karyopherinβ2 (Kapβ2), subclasses of the Kapβ family of proteins, are major NIRs and many transport cargos for Impα/β and Kapβ2 have been identified. RBPs that form SGs, TDP-43 contains a canonical NLS (cNLS), which is recognized by Impα/β. FUS and hnRNPA1 contain a proline-tyrosine NLS (PY-NLS) which is recognized by Kapβ2. TDP-43, FUS, and hnRNPA1 all localize in the nucleus due to their NLS. Disease-related mutations of FUS are concentrated in the PY-NLS region, and accumulation of the nuclear protein in the cytoplasm has been observed in disease onsets. Given that Kapβs can act to dissociate self-assembly of RBPs, this suggests that the regulation performed by Kapβs likely plays an important role in prevention of diseases. It also indicates the presence of additional functions of NIRs aside from their canonical functions which have yet to be explored.

**Fig. 2:**
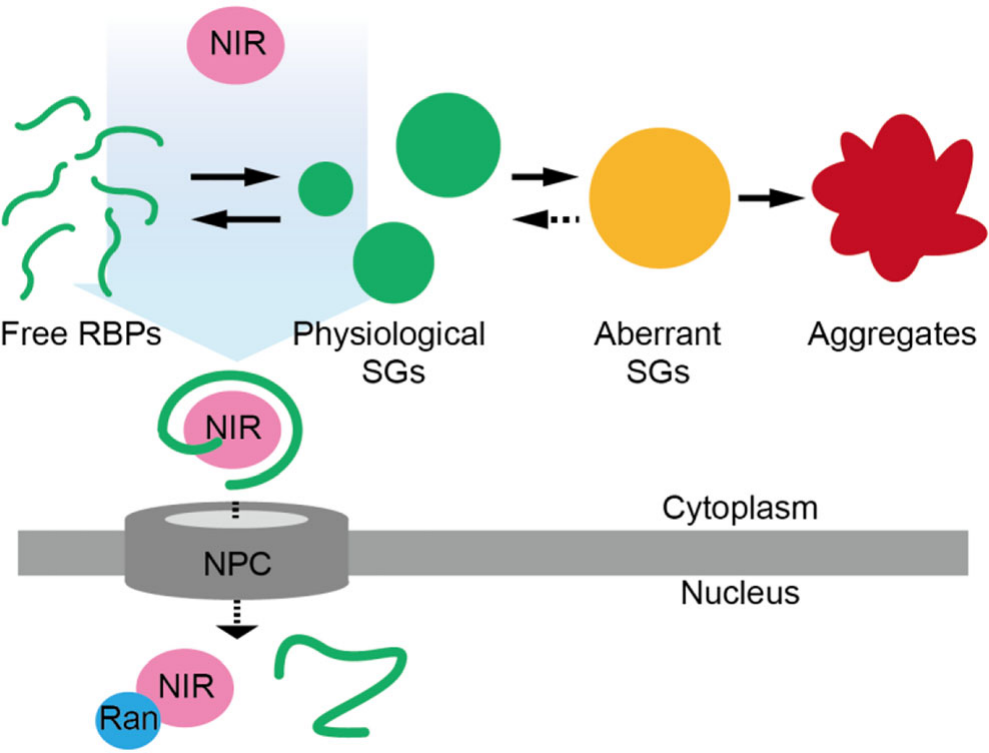
Model of chaperone function of NIRs for RBPs in cells. NIRs (pink) actively carry nascently translated proteins (green lines) or proteins in physiological SGs (green circles) into the nucleus, preventing LLPS from occurring in the cytoplasm. Small GTPase Ran (blue) displaces the proteins and binds NIR in the nucleus. In the absence of this regulatory system, physiological SGs could then subsequently transform into aberrant SGs (yellow) and irreversible aggregates (red) in the cytoplasm

In order to transport cytoplasmic cargos into the nucleus, Kapβs pass through nuclear pore complex, a giant ring protein complex composed of multiple copies of 30 different proteins called nucleoporins. Within the ring complex is a separated phase comprising phenylalanine-glycine repeats (FG-repeats) of FG-nucleoporins. This permeability barrier typically prevents unexpected concentration-dependent diffusion of biological macromolecules between the nucleus and the cytoplasm (Schmidt and Görlich 2016). However, Kapβs are able to penetrate nuclear pore complex by binding with the FG-repeats. One of the current authors and colleagues (Yoshizawa et al. 2018) found that NIRs utilized this function to disassemble LLPS of FUS through Kapβ2, the predominant NIR for FUS. Kapβ2 inhibits and reverses FUS LLPS binding through multiple sites including LC regions that contribute to LLPS. This chaperone function depends on the ability of Kapβ2 to bind to the PY-NLS of FUS. The location of the NLS of each RBP may also play a factor in their assembly/disassembly processes. The PY-NLS of FUS is located at the C-terminal end, while the PY-NLS of hnRNPA1 is located in the middle of the LC domain. Although the LC domain potentially contributes to LLPS through the formation of a cross-β core for hnRNPA1, Kapβ2 can still disassemble hnRNPA1 LLPS structures, likely through direct interactions with the PY-NLS, which are stronger than the LC-contributions to LLPS. However, Kapβ2 cannot disassemble TDP-43 LLPS structures since TDP-43 does not contain a PY-NLS; it instead contains a cNLS, which can be recognized only by Impα/β. Unsurprisingly, Impα/β shows chaperone function for TDP-43 but not for FUS and hnRNPA1, but interestingly, Impα/β can inhibit LLPS of chimeric FUS, where its PY-NLS has been replaced by a cNLS at the C-terminus. This suggests that as long as the NLS of a given SG-forming protein can be identified by the proper chaperone protein, chaperone activity and subsequent LLPS structure disassembly can occur. Taken together, a tight and specific interaction between NLS and NIR is required to inhibit LLPS of SG-forming RBPs. When Yoshizawa et al. (2018) performed further investigations into the relationship between FUS and Kapβ2 using NMR, they detected weak interactions between the exterior of the FUS PY-NLS and Kapβ2. This result suggests that tight binding to NLS by Kapβ2 allows Kapβ2 access to the area of the protein, which contributes to its LLPS character and results in its disassembly. Cellular model experiments also revealed that Kapβ2 expression blocks FUS accumulation in SGs. As such, the chaperone function of NIRs toward RBPs is likely important regulatory processes used by the cell to dissociate SGs in physiological conditions (Fig. 2). Nonetheless, once any of these processes become aberrant, uncontrolled aggregation of SGs may occur, leading to a variety of diseases.

Recent studies have uncovered mechanisms of regulation of SGs in both in vitro and in vivo systems, suggesting that SGs utilize the dynamic and controllable properties of LLPS to protect cells. Understanding the regulation of cellular LLPS sheds new light on our understanding of SGs. However, there is still a large gap between in vitro and in vivo studies. For example, in vivo SGs contain a number of proteins and RNAs, and the location and means by which these molecules are segregated and compartmentalized remain unclear. Post-translational modification and other chaperones are also likely important to regulate SG states.

## A new view of chromatin behavior

In higher eukaryotes, DNA is packaged into chromatin that is organized as chromosomes in the nucleus (Gilbert 2019). The organization and regulation of chromatin architecture has been studied for many years, because chromatin structure plays an important role in gene expression and genome integrity. Indeed, the alteration of chromatin structure or stability underpins many human genetic diseases and cancer.

**Fig. 3:**
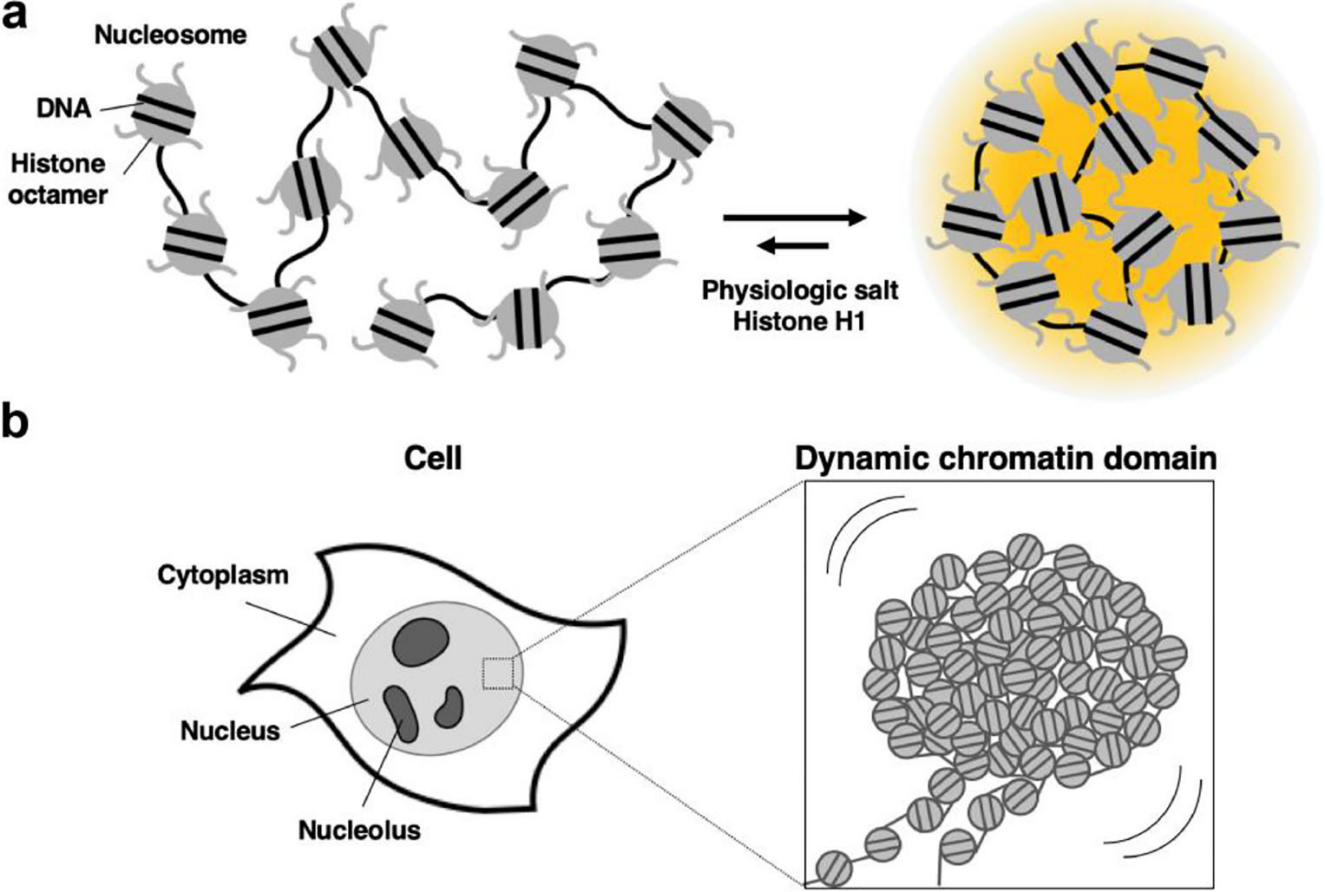
A new view of chromatin behavior. **a** A nucleosome array forms phase-separated condensate in physiological salt concentrations facilitated by histone H1 (Gibson et al. 2019). **b** Visualizing individual nucleosomes using live-cell superresolution imaging technique suggests chromatin forms a large irregular structure behaving like “liquid drops” in the cell (Nozaki et al. 2017)

The fundamental structural unit of chromatin is the nucleosome, which consists of negatively charged DNA containing a phosphate backbone wrapped around an octameric assembly of positively charged histone proteins (Kornberg and Lorch 1999). It is predicted that only approximately half of the negative charges of the DNA are neutralized by the positive charges of the histones (Khrapunov et al. 1997; Schiessel 2003; Maeshima et al. 2014). In fact, early experiments demonstrated that free cations promote self-association of chromatin (Hansen 2002); this is a crucial insight into the properties of chromatin. This process seems to leave some room for free cations and positively charged proteins, such as linker histones, to modulate local chromatin structure by binding chromatin in nucleosomes. Very recent observations indicated that reconstituted chromatin undergoes LLPS in solutions containing physiological cation concentrations (Fig. 3a, (Gibson et al. 2019)). The interaction between DNA and the histone tail enriched with positively charged amino acids drives the LLPS. In addition, LLPS is facilitated by linker histone H1, a result which is in line with early observations that histone H1 depletion causes chromatin unfolding (Allan et al. 1981) and recent biophysical approaches showing that H1-DNA forms a condensate (Turner et al. 2018). Further analysis showed that the acetylation of histone resulted in dissolution of chromatin condensates, while acetylated chromatin was triggered to re-phase-separate by transcriptional regulator bromodomain-containing protein 4 (BRD4), which recognizes the acetylated histone tail. Intriguingly, two distinct-phase, unmodified chromatin condensates and newly induced condensates adhered to each other but did not coalesce, suggesting LLPS is involved in organizing functionally distinct but physically adjacent chromatin domains in the nucleus.

The Maeshima laboratory recently provided a new view of chromatin behavior in the cell using live-cell super-resolution imaging (Nozaki et al. 2017). Their single nucleosome tracking analysis showed that nucleosomes form domains approximately 160 nm in diameter that behave coherently in live cells (Fig. 3b). They suggested that the behavior of chromatin domains is like “liquid drops” rather than static and regular solid-like structures more physically constrained in nucleus. This observation led us to speculate that chromatin domains are phase-separated through self-assembly inside the nucleus and that it may provide chromatin with platform plasticity so that chromatin can conduct its many functions such as gene expression, DNA repair, and cell cycle-specific matters.

## Modulating chromatin structure—compaction and decompaction

Heterochromatin is defined as a chromatin segment that is highly condensed throughout the cell cycle. It is known that abnormally rearranged heterochromatin epigenetically represses the expression of nearby genes. The molecular mechanisms for chromatin condensation and propagation of gene silencing in heterochromatin have been studied for many years (Allshire and Madhani 2018).

**Fig. 4:**
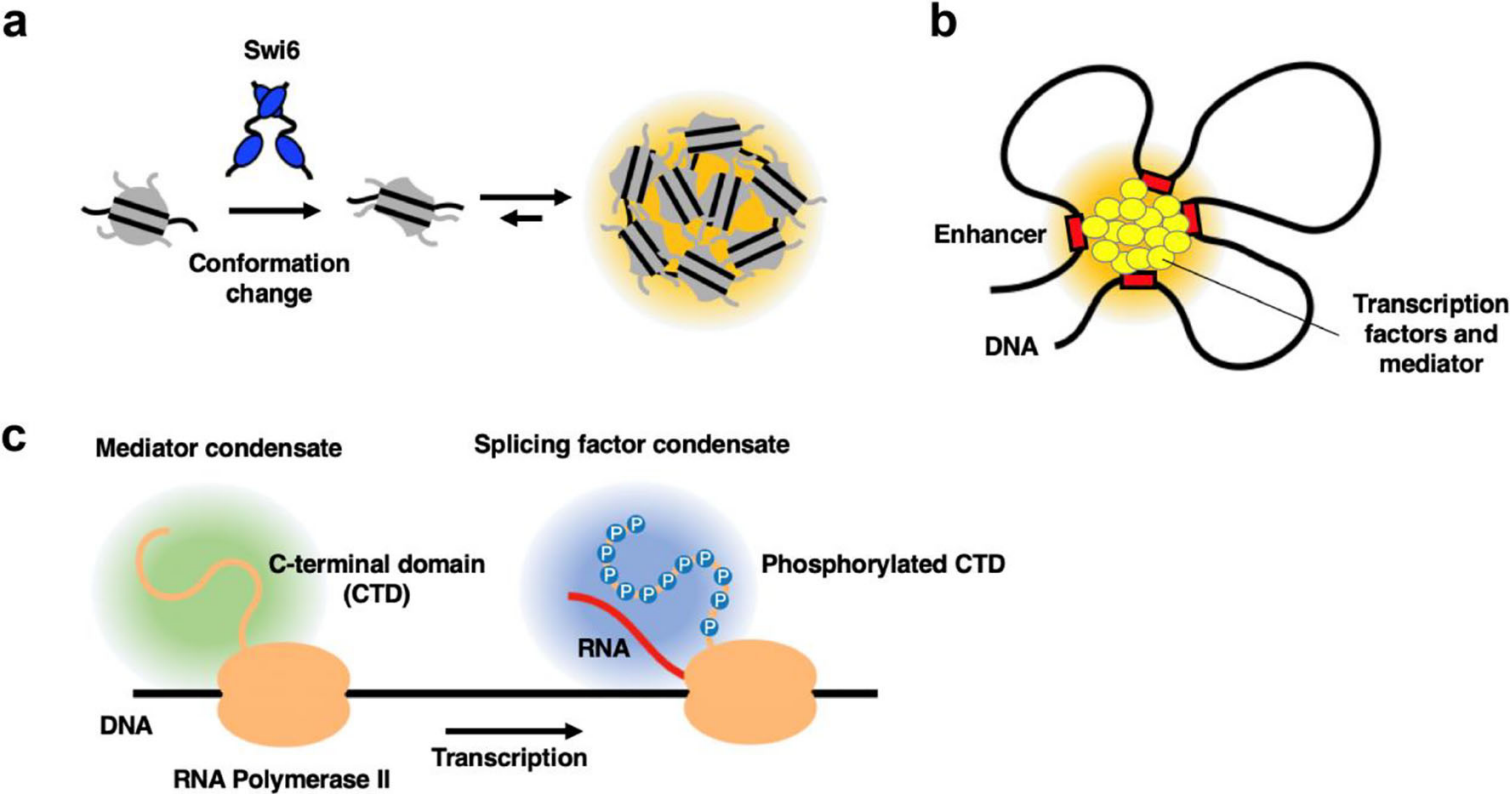
The distinct microenvironment on chromatin driven by LLPS. **a***Schizosaccharomyces pombe* HP1 protein Swi6 induces a conformation change of nucleosome, resulting in the formation of condensed, highly concentrated nucleosomes (Sanulli et al. 2019). **b** Super-enhancers are a cluster of enhancers bound by transcription factors and a mediator that concentrate to create a highly transcriptionally active genomic region (Hnisz et al. 2017). **c** Phosphorylation of the C-terminal domain (CTD) of RNA polymerase II switches its affinity from the mediator to splicing factors (Guo et al. 2019)

Heterochromatin protein 1 (HP1) was identified as a major component of heterochromatin. HP1 recognizes the trimethylation of histone H3 lysine 9 (H3K9me3), a hallmark of heterochromatin, through binding of the N-terminal chromodomain (CD), and dimerizes through binding of the C-terminal (carboxy-terminal or COOH-terminal) chromoshadow domain (CSD) (Brasher 2000; Lachner et al. 2001). The dimerization of HP1 creates a hydrophobic surface that binds to a number of proteins containing the PxVxL (where P is proline, V is valine, L is leucine, and x is any amino-acid residue) motif (Nozawa et al. 2010). A recent report suggested direct evidence that HP1 facilitates chromatin compaction (Sanulli et al. 2019). The CSD of the *S. pombe* HP1 protein, Swi6, interacts with the nucleosome core through the α-helix of histone H2B containing a PxVxL motif. The interaction disorganizes the conformation of the histone octamer, which allows the Swi6-nucleosomal array to efficiently form phase-separated liquid condensates (Fig. 4a). This leads to a higher concentration of nucleosome within the condensates. These results suggest that Swi6 increases opportunities for multivalent interactions between nucleosomes, implying that Swi6 compacts chromatin into liquid condensates. Previous reports suggest that the mechanism of chromatin compaction would be variable among different species. The CSD of mammalian HP1 has been shown to interact with the histone H3 of the nucleosome core but not histone H2B. Additionally, human HP1α and *Drosophila* HP1a themselves were reported to undergo phase separation (Larson et al. 2017; Strom et al. 2017). The LLPS structures of human HP1α required the phosphorylation of the disordered N-terminal region or DNA binding, while the LLPS of *Drosophila* HP1a did not require such phosphorylation.

It is still unknown how large-scale chromatin structures are compacted. Recent reports give us useful insights to consider this question. A polymer-modeling simulation suggested that chromatin assembly can be induced by bridges between nucleosomes as bridging nucleosomes increases the number of chances to create additional bridges on proximal nucleosomes. This “bridging-induced attraction” or “polymer-polymer phase separation” drives a type of de-mixing of the chromatin polymer (Brackley et al. 2013; Erdel and Rippe 2018). Additionally, the CasDrop technology, i.e., inducing an LLPS on chromatin by combining clustered regularly interspaced short palindromic repeats (CRISPR) and optogenetics, demonstrated that condensates induced on targeted chromatin pull targeted loci together (Shin et al. 2018). These results lead us to imagine that HP1-nucleosome condensates play a central role in the “spreading” of heterochromatin to large-scale chromatin structures. Another recent chromatin modeling study combined with Hi-C (high-throughput chromosome conformation capture) analysis suggested that interaction of heterochromatic regions, but not euchromatic regions, leads to compartmentalization of heterochromatin and euchromatin (Falk et al. 2019).

If chromatin compaction progresses in the self-organizing manner as described above, active systems to decompact chromatin would also be required for chromatin compartmentalization. Nozawa et al. have shown that scaffold attachment factor A (SAF-A) decompacts transcriptionally active large-scale chromatin structures (Nozawa et al. 2017). SAF-A was originally identified as a component of the nuclear matrix and was simultaneously found as a member of the family of heterogeneous nuclear ribonucleoproteins (hnRNPs). SAF-A is a highly abundant protein and was observed to be enriched in the transcriptionally active region. Nozawa et al. found that SAF-A has an oligomerization activity that is required for chromatin decompaction. SAF-A’s oligomerization is ATP dependent through SAF-A’s ATPse domain, and it binds RNA through its RG/RGG (where R is arginine and G is glycine) motifs. It seems that SAF-A/RNA filaments biochemically form a nuclear mesh in the nucleus. Nozawa and Gilbert (2019) suggest that interactions between SAF-A and RNA form a transcriptionally responsive dynamic nuclear mesh that creates a high viscosity microenvironment keeping chromatin decompacted.

## Distinct microenvironment on chromatin

The Hi-C technique, a method for quantifying pairwise chromatin interaction frequency genome-wide, revealed that the genome is organized in distinct compartments at the megabase-scale. In other words, transcriptionally active A compartments and inactive B compartments were identified (Lieberman-Aiden et al. 2009). Further studies showed that the existence of A/B compartments was consistent with replication timing (Dileep and Gilbert 2018; Takahashi et al. 2019). The molecular mechanisms responsible for compartment formation and its significance are still unclear but it seems that the organization of large-scale chromatin structure is crucial for creating distinct microenvironments to regulate chromatin functions.

It is also becoming clear that LLPS is a driving force that creates a microenvironment to facilitate chromatin functions. Super-enhancers were proposed as distinct genome regions where several enhancers, transcription factors, mediators, and RNA are locally clustered and were explained to be mediated by LLPS (Fig. 4b; Hnisz et al. 2017). In fact, RNA polymerase II (pol II), transcription factor FET (FUS/EWS [Ewing sarcoma breakpoint region 1]/TAF15 [TATA-binding protein-associated factor 2 N]) family proteins, mediator complex subunit 1 (MED1), and BRD4 were shown to form condensates through LLPS and all were observed to form clusters in the nucleus (Boehning et al. 2018; Chong et al. 2018; Sabari et al. 2018). The interaction among these clusters was highly dynamic and selective (Cho et al. 2018). Another crucial observation suggested that cluster formation and interaction among clusters are regulated by phosphorylation (Kwon et al. 2013; Boehning et al. 2018; Guo et al. 2019). For example, the C-terminal domain (CTD) of pol II is a disordered low-complexity region that consists of the repetitive heptad amino acid sequence (Tyrosine, Serine, Proline, Threonine, Serine, Proline, Serine). The CTD is known to be stepwise phosphorylated in transcription processes such as initiation, elongation, and termination. The Young laboratory demonstrated that CTD phosphorylation facilitates its condensate’s preference switch between MED1 and the splicing factor condensate (Fig. 4c**,** (Guo et al. 2019)). Altogether, these recent reports suggest that pol II CTD plays a major role as a hub to create a chromatin microenvironment by selectively associating with transcriptional factors. The association is dynamically regulated by stepwise phosphorylation of the CTD. Contributions of LLPS to regulation of not only transcription but also DNA replication (Parker et al. 2019), DNA damage repair (Kilic et al. 2019), and mitotic progression (Trivedi et al. 2019) have also been observed, suggesting that a number of biological processes likely are conducted in spatiotemporally assembled phase-separated microenvironments.

It should also be mentioned that RNA has various characteristics that potentially affect chromatin functions through LLPS. First, as described above, RNA serves as a seed for biomolecular assemblies. Not only does rRNA serve as a seed, but the long non-coding RNA (lncRNA) Nuclear Enriched Abundant Transcript 1 (NEAT1) acts as a seed by recruiting specific proteins, subsequently forming distinct compartments called paraspeckles (Hirose et al. 2019). NEAT1 location is simultaneously defined because the assemblies form spatially near the locus from which they are transcribed. X-inactive specific transcript (XIST) RNA, another lncRNA, has also been proposed to drive LLPS to create silent compartments for X chromosome inactivation (Cerase et al. 2019). Next, RNA buffers LLPS processes, suggesting a regulatory aspect of RNA-base LLPS systems (Maharana et al. 2018). The Hyman laboratory demonstrated that FUS, a RBP containing an LC domain, specifically forms condensates with NEAT1 while FUS condensates dissolve with higher concentrations of non-specific RNAs, suggesting that LLPS of FUS is controlled by both specific and non-specific RNAs. Moreover, the RNA itself also undergoes LLPS (Jain and Vale 2017). Expansion of short nucleotide repeats is found in several neurological and neuromuscular disorders. A transcript containing such repetitive sequences drives self-assembly through multivalent base-paring with a similar repeat number as observed in the disorders, resulting in a potential disruption of cellular functions. Finally, RNA acts as “glue” to bind various molecules and stably localize them to the same location (Ding et al. 2019). A recent compelling study in yeast demonstrated that several meiosis-specific lncRNAs and their binding proteins form condensates via LLPS on several specific loci. These condensates assist with chromosome pairing by their fusion, creating a link between homologous chromosomes at the prophase stage of meiosis. This lncRNA-based mechanism defines pairing specificity of homologous loci, preventing undesired pairing. Mouse X chromosomes were also observed to be transiently paired during X chromosome inactivation, a stochastic process in which a given chromosome may be inactivated with a certain probability (Masui et al. 2011). Some evidence suggests that the antisense repressor of XIST (Tsix) RNA, the antisense strand of XIST RNA, is involved in choosing whether the X chromosome is inactivated. This observation leads us to imagine a similar system where chromosome pairing was first developed in yeast, and then the process was conserved even upon evolution to eukaryotic cells.

## LLPS processes in the origin of life

Modern cells, ubiquitous to all life, are composed of various organelles built from assemblies of biomacromolecules. Such cells generally contain a cell membrane (usually a phospholipid bilayer also containing various cholesterols and proteins), a genetic material (DNA that can grow, replicate, and divide), and biochemical reactions which drive processes such as metabolism and replication. However, how such a cell could have emerged and evolved is still an open question. One proposal is that modern cells arose from a more primitive form of compartmentalization on early Earth (i.e., a protocell) which provided a primitive system the ability to concentrate (Keating 2012; Aumiller and Keating 2016) and segregate reactants (Jia et al. 2019). These compartments would have protected molecules from environmental degradation (Shirt-Ediss et al. 2017) and provided a segregated location to allow evolution of genetic replicators without being overtaken by parasites (Bansho et al. 2016). While these could have been some of the general functions of primitive compartments, there are other more nuanced requirements for a compartment to be considered prebiotically plausible or relevant, such as the ability to allow nutrients to enter and byproducts to exit the compartment easily, as well as the ease of synthesis through prebiotically plausible mechanisms (Szostak et al. 2001). Fatty acid bilayer membrane vesicles (Mansy and Szostak 2009) are an attractive model for protocells due to their ability to encapsulate important primitive molecules such as RNA as well as their permeability to small nutrients necessary for prebiotic processes (Luisi et al. 1999; Chen and Walde 2010; Budin et al. 2014). Fatty acid membrane-based vesicle compartments which have life-like functions such as growth (Adamala and Szostak 2013a; Hentrich and Szostak 2014), replication (Adamala and Szostak 2013b), and division (Zhu and Szostak 2009; Zhu et al. 2012) have been demonstrated. While these findings appear to support such systems as the preferred prebiotic cellular framework, challenges still persist with regard to the prebiotic synthesis of some fatty acids, especially when compared with other prebiotic molecules such as amino acids, peptides, nucleosides, and, until very recently, assembly of simple vesicles composed of mixtures of fatty acids (Jordan et al. 2019). While it is clear that a lipid bilayer membrane-containing compartment was, at some point, likely the precursor of a modern cell, exactly when and how such a membrane-based primitive cell emerged is still unknown. It is possible that other forms of primitive compartmentalization preceding (or co-existing with) such membrane-bound compartments may have existed.

One potential prebiotic compartment candidate system that could have been produced on early Earth (for example, from simple peptides, nucleotides, or polyesters) exhibits “life-like” behaviors and shows compatibility with modern biomolecules having structures derived from LLPS of polymeric systems (Mann 2012; Keating 2012; Yin et al. 2016; Matsumura et al. 2016; Jia et al. 2019). In vitro laboratory simulations of LLPS processes result in formation of co-existing liquid (or liquid-like) droplets within a bulk liquid through primarily energetically favorable processes (such as entropic effects and hydrogen bonds; (Alberti et al. 2019)) rather than through production of energy intensive covalent bonds (Pascal and Boiteau 2011). These droplet systems are membrane-less and can be composed of simple heterogeneous polymer systems more closely resembling synthetic products from early Earth, suggesting that LLPS compartments may have been able to form easily on early Earth. As discussed in other sections of this review, LLPS are ubiquitous in biology as subcellular compartments used to segregate and compartmentalize important biomolecular analytes in various biological processes (Lin et al. 2016; Alberti 2017b; Chong et al. 2018). This hints at the presence of a direct link between primitive LLPS systems and those observed in modern biology. In the following sections, we will briefly review some of the major types of prebiotically relevant LLPS systems by highlighting their composition, structure, and functions.

## Aqueous two-phase systems

Aqueous two-phase systems (ATPS) are LLPS systems typically composed of one or more co-existing polymers such as poly (ethylene) glycol (PEG) and dextran. Such ATPS spontaneously separate into two distinct phases depending on the concentration of the components (the higher the concentration of both components, generally the greater the phase separation), the solution conditions, and temperature (Keating 2012; Yanagisawa et al. 2014; Iqbal et al. 2016). Upon agitation (perhaps through primitive geological processes such as wind, tides, or turbulence driven by fumaroles or other pressurized hydrothermal structures; (Chiodini et al. 2012)), the two phases rearrange into membrane-less liquid-in-liquid droplets. In the case of PEG/dextran systems, it forms dextran-rich droplets within a bulk PEG-rich phase up to tens of microns in size (Keating 2012). Over time, the droplets eventually coalesce, and the two phases separate into a droplet-less upper and lower phase. However, re-agitation will re-form the liquid-in-liquid droplet state.

**Fig. 5:**
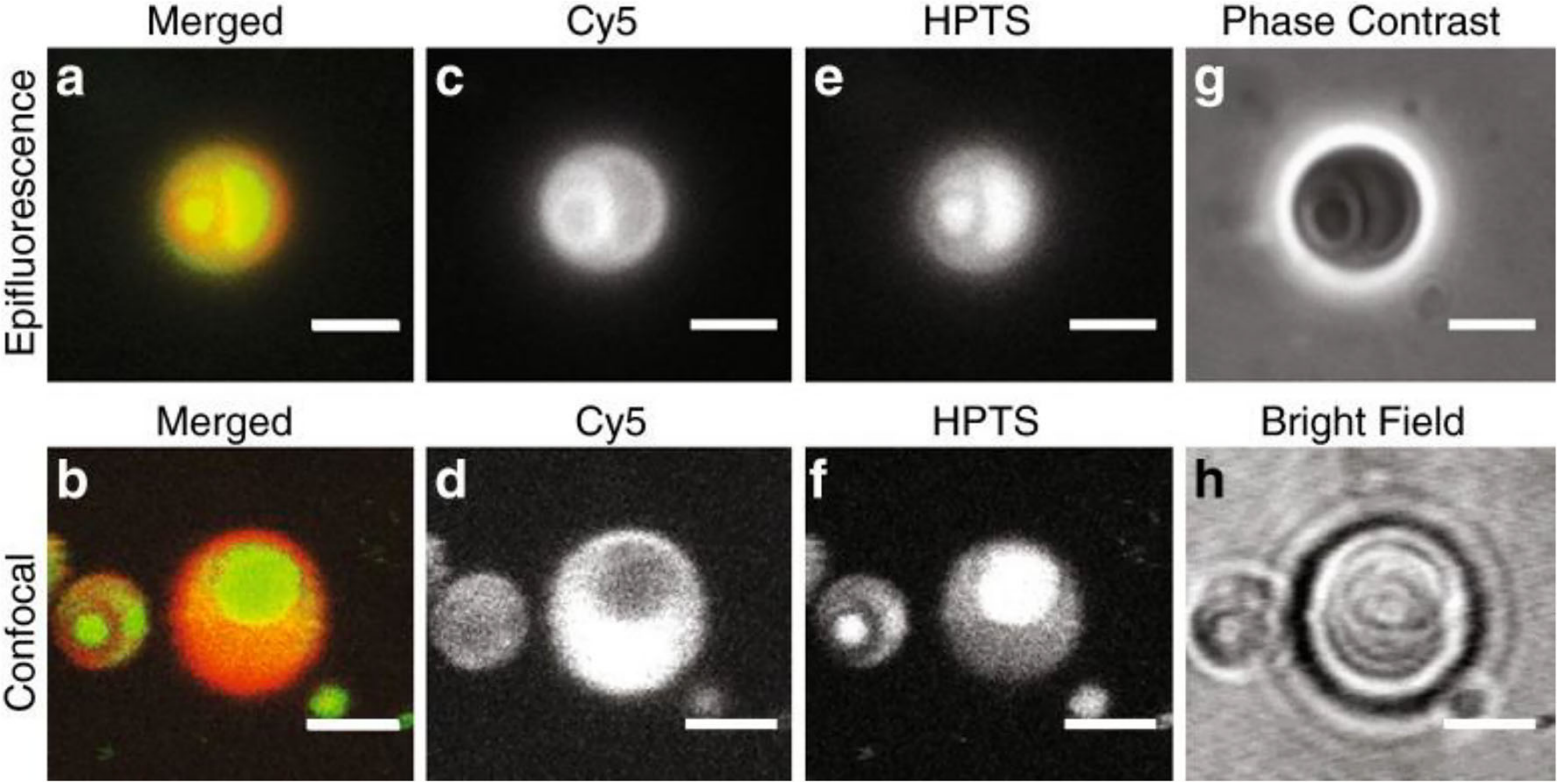
Poly (ethylene) glycol (PEG)/dextran phase-separated droplets encapsulated within oleic acid vesicles. The top row shows images acquired by an epifluorescence microscope, while the bottom row shows images acquired by a confocal microscope. **a**, **b** Merged images of (**c**) and (**e**) or (**d**) and (**f**), respectively, which show separated fluorescence channels. Cyanine 5 (Cy5)-labeled RNA (red in (**a**) and (**b**)) partitioned into the outer dextran phase as shown in (**c**) and (**d**), while HTPS (8-hydroxypyrene-1,3,6-trisulfonate) (green in (**a**) and (**b**)) partitioned into the PEG phase as shown in (**e**) and (**f**). **g**, **h** Phase contrast and bright field images. Image reprinted with permission from Jia, Tony Z., Hentrich, Christian, Szostak, Jack W.: “Rapid RNA Exchange in Aqueous Two-Phase System and Coacervate Droplets.” *Origins of Life and Evolution of Biospheres*, **44**, 1–12 (2014) (Jia et al. 2014) under a Creative Commons License

While assembly of these droplets can be achieved from simple physical means, elucidation of the potential prebiotically relevant functions is still necessary before ATPS systems can be fully considered as primitive compartmentalization mechanisms. Some ATPS systems have been shown to be able to segregate biopolymers such as nucleic acids (RNA; Jia et al. 2014) and peptides (Chu and Chen 2000), resulting in increased analyte concentration (which may drive yield of primitive reactions). However, as the ATPS system does not include a membrane boundary, it would likely allow for free exchange of certain analytes, i.e., nutrients, between droplets depending on the droplet composition, a necessary function in early pre-biological chemical systems. Such systems can also be compartmentalized within lipid bilayer membrane vesicles, simulating a primitive cell-like structure with distinct phases within the structure (Jia et al. 2014) (Fig. 5). Finally, ATPS droplets can scaffold the assembly of a layer of clay minerals, themselves an important prebiotically available geological structure promoting the synthesis and self-assembly of primitive polymers (Gillams and Jia 2018), around the droplet edge (Pir Cakmak and Keating 2017) (similar to a Pickering emulsion (Yang et al. 2017)), helping to catalyze prebiotically relevant chemical reactions and to prevent coalescence of the droplets. The addition of a clay mineral layer (and perhaps a lipid layer as well) serves to preserve each droplet’s individuality (Smith and Morowitz 2016), an important property important for selective evolution of primitive replicating polymers within compartments (Bansho et al. 2016). Although PEG and dextran polymers, the most studied ATPS system in origins of life studies, may themselves not be prebiotically plausible, the ATPS droplet system still provides a model framework which can be used to study the relevant properties of ATPS systems in laboratory simulations. The fact that such membrane-less droplets can be assembled through simple physical processes by other geochemically available means on early Earth and with other prebiotically relevant systems such as oil-in-water or water-in-oil droplets suggests their possible significance as primitive compartments at the origin of life and validates their continued use as model systems to study primitive compartmentalization.

## Coacervate droplets

**Fig. 6:**
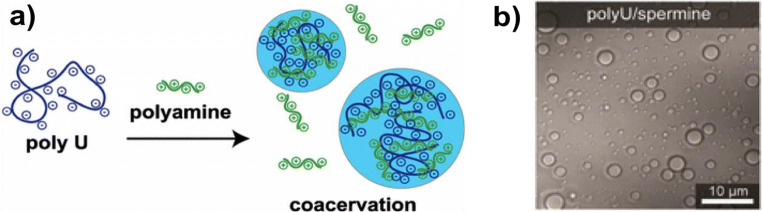
Example of a coacervate droplet system composed of a nucleic acid (polyU RNA) and a simple polyamine (**a**). The negative charges on the RNA backbone bind strongly to the positive charges on the polyamine, resulting in a condensed coacervate phase consisting of both components (blue), and a dilute aqueous phase. **b** A micrograph of coacervate droplets produced from polyU and spermine. Image reprinted with permission from Aumiller, William M., Pir Cakmak, Fatma, Davis, Bradley W., Keating, Christine D.: “RNA-Based Coacervates as a Model for Membraneless Organelles: Formation, Properties, and Interfacial Liposome Assembly” *Langmuir*, **32**(39), 10,042–10,053 (2016), (10.1021/acs.langmuir.6b02499) (Aumiller et al. 2016). Copyright American Chemical Society; further permissions related to the material excerpted should be directed to the American Chemical Society

Coacervate droplets are another membrane-less droplet structure well-represented in extant biology. Protamine (Hud et al. 1994; Balhorn et al. 2000), RNA granules (Kedersha and Anderson 2009), chromatin (Widom 1998), and nucleoli (Weber and Brangwynne 2015) are all examples of modern cellular coacervates, some of which control human disease (Shin and Brangwynne 2017). Such membrane-less coacervate droplets are fundamentally different in nature than ATPS, generally forming from interactions between two or more oppositely charged species (Priftis and Tirrell 2012) such as ATP and poly-lysine (Jia et al. 2014) or RNA and polyamines (Aumiller and Keating 2016) (Fig. 6). Initial phase separation occurs due to the immiscibility caused by a shift in the solvation energy upon binding of the analytes (Veis 2011). However, such phase separation also depends on other factors including temperature, pH, salt concentration, and the length and concentration of the “coacervating” analytes (Priftis et al. 2013). Upon further agitation, potentially caused by primitive geological processes such as wind, tides, or turbulence driven by fumaroles or other pressurized hydrothermal structures (Chiodini et al. 2012), the separated phases assemble into membrane-less droplets composed of a concentrated phase consisting of the bound analytes and a dilute bulk phase which is mostly aqueous.

In vitro-produced coacervates can segregate and compartmentalize important functional biomolecules (retaining their function and/or structure) such as DNA (Martin et al. 2019), RNA (Poudyal et al. 2018, 2019; Drobot et al. 2018), and proteins (Martin et al. 2016) and can scaffold the assembly of lipid layers (Tang et al. 2014). Coacervate droplets could be more permeable to small nutrients compared with fatty acid membrane vesicles and can be formed from fairly simple prebiotic components such as mononucleotides and short peptides, which potentially eliminates the need for abiotic fatty acid synthesis in primitive systems (Frankel et al. 2016). Such permeability may have been more desirable early on during the origins of life; such systems may be utilized by primitive systems to explore more possible chemistries, as opposed to more stable and selective compartments required for later biochemistries. It is also believed that the interior of a coacervate droplet more closely approximates the cellular organization and crowded environment afforded by the modern cytoplasm compared with the interior of a fatty acid vesicle (Tang et al. 2014). As such, it is possible that biological coacervate systems were ancient in origin, and a primitive coacervate could have provided primordial chemical systems a way to segregate molecules and act as a simple microreactors or even as an ancestor to modern cells themselves (Koga et al. 2011; Jia et al. 2014) while also exhibiting tunable “life-like” functions such as division or fusion (Yin et al. 2016) and heterotrophy (Qiao et al. 2017). While some of the in vitro coacervate examples presented here, mostly produced from peptide-peptide, peptide-nucleotide, peptide-nucleic acid, or polyamine-nucleotide interactions, may be composed of some prebiotically implausible components, such coacervates are still a reasonable model system to study and glean information on prebiotically plausible coacervate systems, given that they share similar structures and functions. Recent research has even shown that tunable coacervates can be produced from more prebiotically plausible analytes such as 10- to 15-residue peptides (Koga et al. 2011; Taniguchi et al. 2016; Aumiller and Keating 2016), simple polyelectrolyes like ATP (Koga et al. 2011), or simple polyamines (Aumiller and Keating 2016), perhaps leading the way for origins of life researchers to advance research in such more prebiotically plausible coacervate systems.

## Polyester microdroplets

**Fig. 7:**
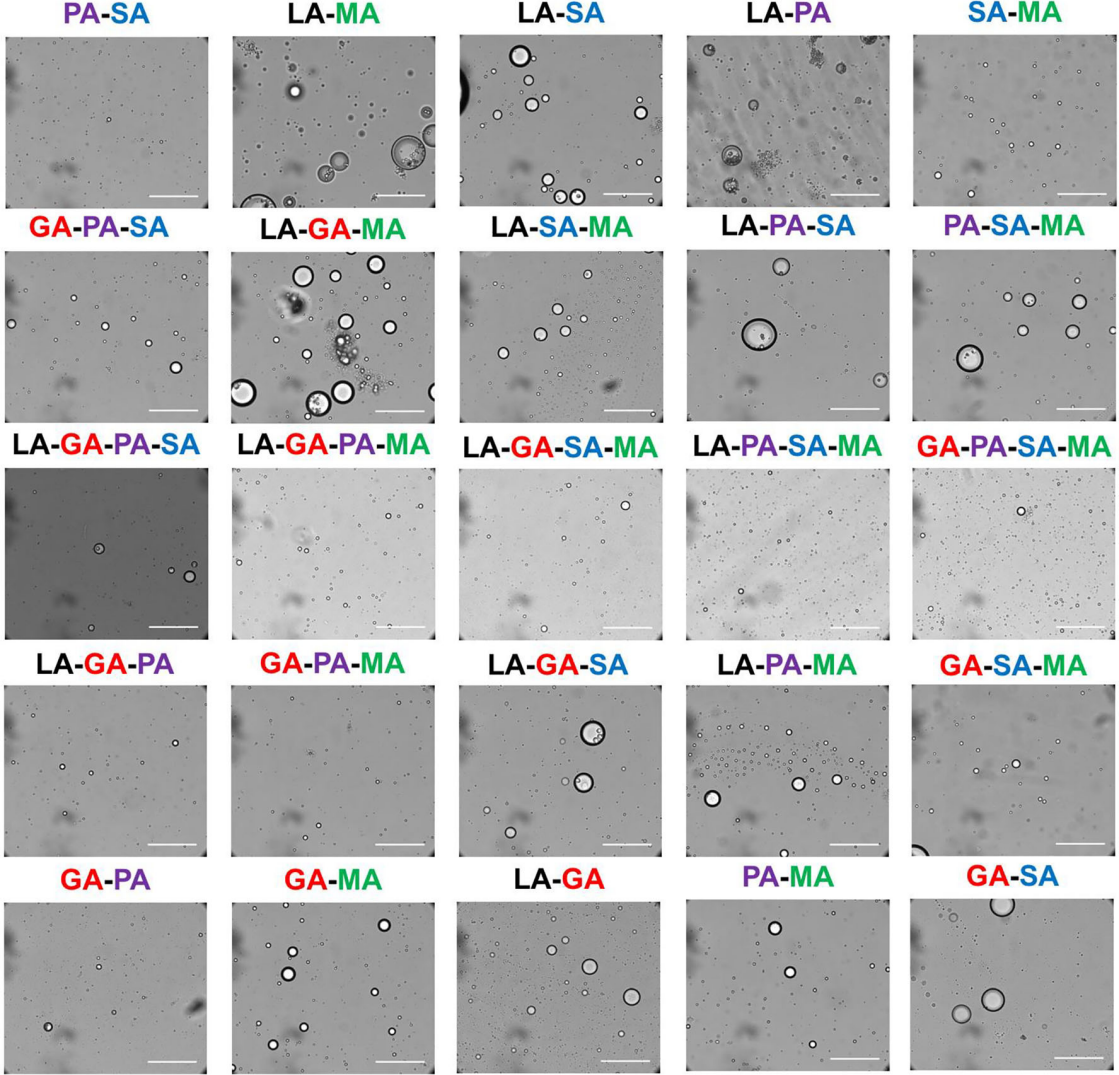
Membrane-less microdroplets assembled from the rehydration of polyesters synthesized by the drying of mixtures of simple, prebiotically abundant alpha hydroxy acids. Scale bars are 100 μm. LA, lactic acid; GA, glycolic acid; PA, phenyllactic acid; SA, 2-hydroxy-4-(methylsulfanyl) butanoic acid; MA, 2-hydroxy-4-methylpentanoic acid. The labels above each image represent the mixture present in that sample. Reprinted with permission from Jia, Tony Z., Chandru, Kuhan, Hongo, Yayoi, Afrin, Rehana, Usui, Tomohiro, Myojo, Kunihiro, and Cleaves, H. James: “Membraneless polyester microdroplets as primordial compartments at the origins of life.” *Proceedings of the National Academy of Science of the USA*, **116**, 15,830–15,835 (2019) (Jia et al. 2019). Copyright Jia, Tony Z., Chandru, Kuhan, et al.

Previous research has shown that the prebiotic milieu contained a large chemical diversity including both biological (e.g., amino acids, peptides, nucleosides) and non-biological compounds of varying complexity (Guttenberg et al. 2017; Walker et al. 2017). Although much origins of life research has focused on such biological molecules, including those with fairly difficult prebiotic syntheses (Li et al. 2017; Gillams and Jia 2018; Milshteyn et al. 2018; Becker et al. 2019), simple non-biological molecules abundant in such primitive chemical pools likely also directly participated in the initial emergence of these first evolving chemical systems (Forsythe et al. 2015; Chan et al. 2019). One such abundant prebiotic non-biomolecule type is alpha hydroxy acids (αHAs), a simple monomer which can be produced from prebiotically plausible mechanisms such as terrestrial spark discharge (Parker et al. 2016) or on extraterrestrial bodies such as on meteorites (Peltzer and Bada 1978). αHAs are very similar in structure to α-amino acids, differing only in replacement of one amino group with a hydroxyl group, and can produce long polyester polymers via simple drying reactions (Chandru et al. 2018). Once polymerization occurs, rehydration results in assembly of a membrane-less microdroplet (Fig. 7). These microdroplets can act as primitive compartments, as shown through the segregation of various small molecule dyes, RNAs, and proteins. However, the amount of free analyte exchange (stable compartmentalization) afforded varies greatly depending on the droplet chemistries and the analyte in question. In fact, protein function within and catalytic RNA (ribozyme) function in the presence of the polyester microdroplets are not inhibited and lipid layers can be scaffolded around the droplets, suggesting that these compartments are compatible with modern biologies. Mixed non-biological/biological hybrid compartment systems incorporating LLPS structures, which themselves may have been more accessible in “messy” prebiotic environments, will be the next goal in origins of life research as they may have the potential to result in emergent properties including protection, exchange, and encapsulation of primitive components. The fact that a membrane-less compartment can be assembled from prebiotically relevant reactions of primitive molecules importantly gives evidence of the potential of a direct pathway from the “messy” prebiotic milieu into a functional primitive compartment.

## Techniques to observe structure and dynamics of phase-separated proteins

Next, we summarize the techniques typically used for investigation of the structure, interactions, and dynamics of LC proteins in LLPS droplets. Although some cellular LLPS droplets and organelles contain other components, such as nucleic acids, in addition to proteins or even no proteins at all (in vitro ATPS and polyester systems), we focus here on the techniques that target proteins, as most cellular LLPS droplets and organelles contain at least one protein component. Light and fluorescence microscopy are frequently used to observe droplets. For example, fluorescence recovery after photobleaching (FRAP) assays evaluate the liquid property of droplets, and time-lapse imaging evaluates merging and coalescence kinetics of the droplets (Li et al. 2012). Digital holographic microscopy is another potential method that can be used for 3D profiling and tracking of the LLPS droplets (Bolognesi et al. 2011; Marquet et al. 2013). Although microscopic observations of LLPS droplets provide much information about the nature of the droplet, more detailed “molecule-level” information is required to unveil the mechanism of the formation and regulation of such droplets. Given the dynamic nature as well as the heterogeneity of proteins within LLPS droplets, probing these structures is not straightforward. Here, we highlight several key techniques used to investigate the proteins in LLPS droplets at molecular and atomic resolution.

## Solution nuclear magnetic resonance

**Fig. 8:**
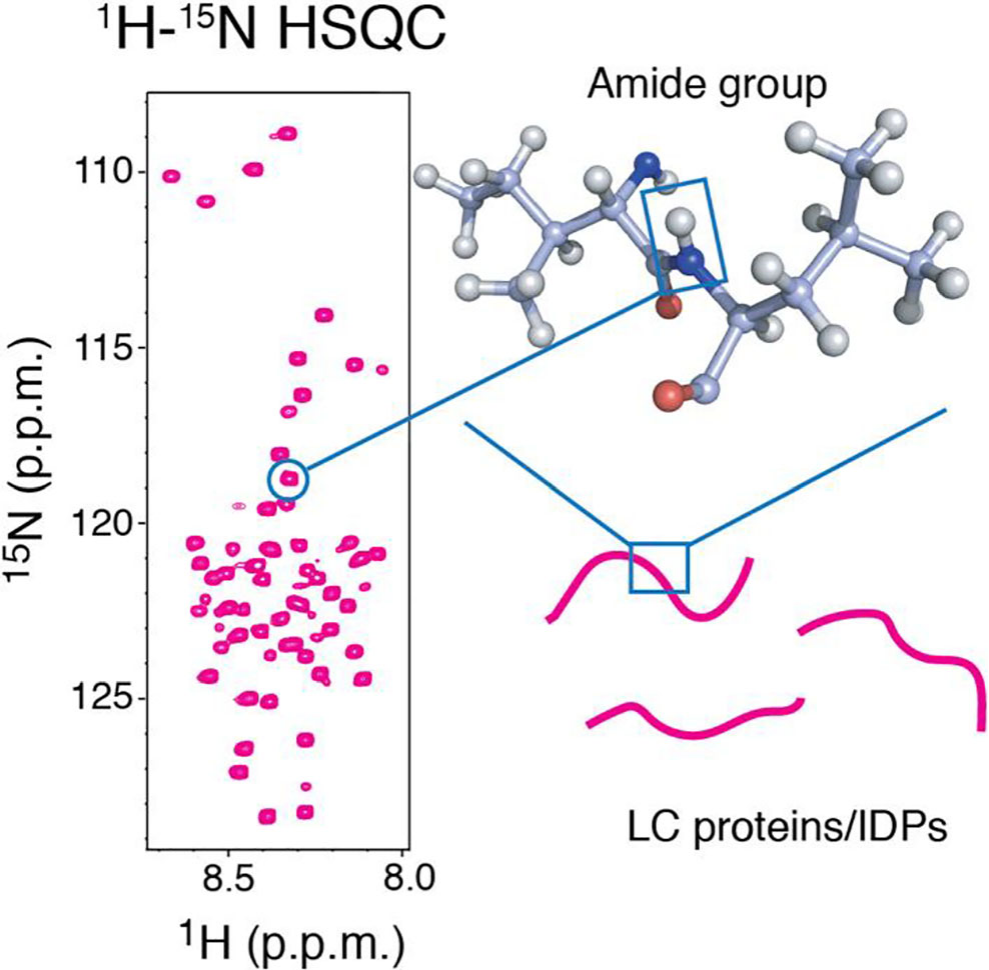
^1^H-^15^N Heteronuclear single quantum coherence (HSQC) spectrum of a disordered protein. In principle, one amide group gives one resonance in the spectrum. If the protein exists in multiple conformational states, each of the conformational states provides distinct sets of resonances. The spectrum of LC domain proteins/IDPs typically shows a narrow chemical shift dispersion

Solution nuclear magnetic resonance (NMR) is one of the most powerful tools used to investigate the structure and dynamics of protein in solution at atomic resolution. In protein solution NMR, the resonances of ^1^H, ^15^N, and ^13^C are frequently observed due to their presence in protein structures, among which ^15^N and ^13^C must usually be enriched by the use of isotopically labeled reagents during protein expression. The use of the appropriate isotope labeling scheme enables the observation of the specific atoms in the protein. The most common experiment is 2D ^1^H-^15^N HSQC (heteronuclear single quantum coherence spectroscopy), which observes the resonances of the backbone amide group of the protein. Given that all amino acids except proline contain an amide group, the ^1^H-^15^N HSQC spectrum can be a “fingerprint” of the protein at the single-residue level (Fig. 8). A number of HSQC-based experiments investigated the interaction, structure, and dynamics of LC proteins including hnRNPA2 (Ryan et al. 2018) and FUS (Burke et al. 2015; Murthy et al. 2019), as well as other proteins in LLPS droplets (Conicella et al. 2016; Brady et al. 2017; Tsang et al. 2019). These studies reported that all such proteins have predominantly disordered conformations in LLPS droplets.

The interior of an LLPS droplet is highly viscous, and the diffusion of the FUS LC domain in the LLPS droplet was reported to be ~ 500 times slower than in the diluted phase (Murthy et al. 2019). Such restricted molecular motion is disadvantageous to high-resolution solution NMR. Multiple interactions between proteins can induce exchange broadening of the resonances, which further reduces the spectral sensitivity. So far, relatively sensitive NMR resonances of proteins in LLPS droplets have been reported (Burke et al. 2015; Conicella et al. 2016; Brady et al. 2017; Ryan et al. 2018; Tsang et al. 2019; Murthy et al. 2019). Typically, the dynamic nature of the LC proteins that are devoid of a stable tertiary structure is advantageous to high-sensitivity NMR observations. However, it should be noted that in NMR, smaller proteins (such as monomers) provide sharper signals, while larger species such as oligomers provide broad signals which are sometimes easily overlooked in the spectra. Especially in the case of heterogeneous conditions, such as in LLPS droplets, the possibility of the protein size-dependence of the NMR resonances must be considered. The use of NMR experiments that monitor the mobility of the protein, such as pulsed-field gradient (PFG) NMR, can assist in LLPS droplet protein analyses (Murthy et al. 2019); PFG NMR allows the separation of the resonances according to the difference in the translational diffusion coefficients (Pagès et al. 2017). In principle, PFG NMR is able to distinguish the different oligomeric species, but still have difficulty detecting the larger species due to resonance broadening.

In order to increase the spectral sensitivity for large oligomers and/or proteins in a viscous LLPS droplet, techniques utilizing isotope labeling are beneficial. In NMR of slow-tumbling molecules, the transverse relaxation time of the observed nuclei becomes too short to obtain high-resolution NMR spectra, which is one of the current limits of NMR analysis of LLPS proteins. Labeling techniques exploiting ^2^H reduce the transverse relaxation time of the observed nuclei, and thus extend the target protein size limit for NMR observation. Exploitation of methyl-selective isotope labeling combined with methyl-transverse relaxation optimized spectroscopy (TROSY) enabled researchers to obtain high-resolution NMR spectra of the 1 MDa proteasome (Mainz et al. 2013). In methyl-selective labeling, the methyl groups of Ile, Leu, Val, Met, Ala, and Thr are specifically labeled as ^13^C^1^H_3_; other hydrogens in the protein are deuterated, resulting in prolonged transverse relaxation times which provide sharper resonance peaks even for large proteins (Saio et al. 2014). Although the rotational correlation time of the 1 MDa proteasome (rotational correlation time, *τ*_*c*_ 590 ns) is much longer than that of small proteins (e.g., *τ*_*c*_ ~ 15 ns for a ~ 25 kDa grobular protein) and cannot be observed in typical NMR conditions, methyl-based NMR techniques effectively enable high-resolution NMR analysis. These NMR techniques will be beneficial in the future in the analysis of polymers of the LC proteins and LLPS chaperones such as Kapβ2 (100 kDa) (Yoshizawa et al. 2018).

**Fig. 9:**
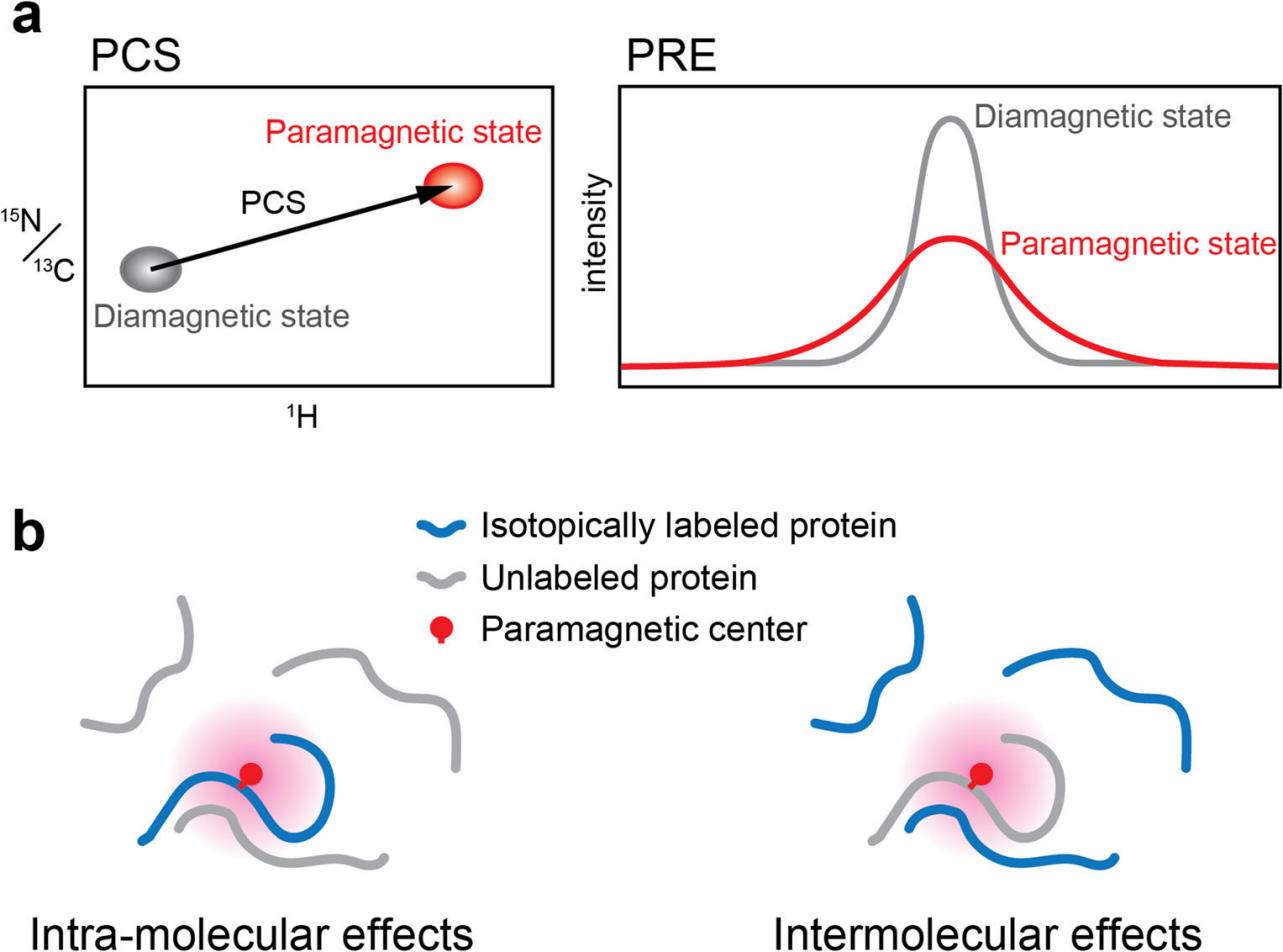
Paramagnetic effects for NMR structural studies on LC proteins. **a** Schematic representation of the paramagnetic effects frequently used in protein structural studies by NMR. Pseudocontact shifts (PCSs) are generated by anisotropic paramagnetic ions such as lanthanide ions, while paramagnetic relaxation enhancements (PREs) are generated by nitroxide spin labels as well as lanthanide ions. **b** Observation of intermolecular and intramolecular paramagnetic effects. When the paramagnetic center is attached to an isotopically labeled protein, observed paramagnetic effects represent intramolecular information (*left*). When the paramagnetic center is attached to an unlabeled protein, observed paramagnetic effects represent intermolecular information (*right*)

Paramagnetic probes are of great use in NMR structural analysis of LC proteins in LLPS droplets. A paramagnetic center, such as a nitroxide spin label, and paramagnetic metal ions including trivalent lanthanide ions can be introduced to the protein of interest via a disulfide bond through a cysteine residue (Saio and Ishimori 2019). The benefit of paramagnetic probes is their ability to assist in observation of long-range structural information of the observed nuclei in the protein. The paramagnetic center induces a variety of paramagnetic effects including paramagnetic relaxation enhancement (PRE) and pseudocontact shift (PCS). PREs provide distance information of nuclei within the range of < 25~30 Å from the paramagnetic center, and PCSs provide distance and angular information of nuclei within the range of < ~ 40 Å (Fig. 9). Intramolecular and intermolecular long-range structural information is beneficial to infer the conformation and oligometric state of LC proteins. For example, when a paramagnetic center is introduced to an isotopically labeled protein mixed with an excess of unlabeled protein, the observed paramagnetic effects provide intramolecular information (i.e., the conformational state of the protein). When a paramagnetic center is introduced to an unlabeled protein mixed with an excess of isotopically labeled protein, the observed paramagnetic effects provide intermolecular information (i.e., the oligomeric state and interaction) (Fig. 9). While the use of PRE has been used in NMR-based structural analyses of LC proteins (Conicella et al. 2016; Ryan et al. 2018; Murthy et al. 2019), other paramagnetic effects have not yet been utilized. Exploitation of the other available paramagnetic effects including PCS and residual dipolar coupling (RDC) will further extend the possibilities of and increase the gleanable information from NMR studies of proteins in LLPS droplets.

## Electron paramagnetic resonance

In addition to NMR, electron paramagnetic resonance (EPR) has been used to monitor the dynamics of proteins in LLPS droplets (Babinchak et al. 2019). The mobility of the site-directed spin label (SDSL) attached to specific positions of the LC protein is evaluated on basis of the EPR spectra. Since EPR signals are highly sensitive to local motions of the paramagnetic spin label attached to the protein, the EPR spectra provide information about protein dynamics. Based on this, the oligomerization of the transactive response TDP-43 in LLPS droplets was investigated (Babinchak et al. 2019). Further developments in EPR techniques of late include distance measurement by pulsed EPR, double electron-electron resonance (DEER) in which the distance and population between paramagnetic centers can be evaluated (Jeschke 2012). With DEER measurements, the structure of the oligomeric species of LC proteins in LLPS droplets can be further investigated. Given this recent development and additional expected developments of EPR techniques, further application of EPR toward LLPS analysis is expected and needed.

## Other biochemical and biophysical methods

In addition to spectroscopic tools, other methods to study proteins within LLPS droplets have been applied. A biochemical method exploiting chemical modification and mass spectroscopy, termed chemical footprinting, which allows identification of the structure of LC proteins in LLPS droplets was conducted (Xiang et al. 2015). Chemical footprinting utilizes N-acetylimidazole (NAI) as a reactive chemical compound that acetylates the side chains of Ser, Tyr, Lys, Thr, Arg, and Asn. By evaluating the residue-by-residue NAI reactivity by stable isotope labeling by/with amino acids in cell culture (SILAC) mass spectrometry, the exposure of each amino acid residue within an LLPS structure to solution can be estimated. Using this method, the existence of the cross-β polymer of the hnRNPA2 LC domain in LLPS droplets was discovered (Xiang et al. 2015).

High-speed atomic force microscopy (AFM) is another promising future tool that can be used to investigate LC proteins. AFM surveys the structure of a protein weakly immobilized onto a sample stage substrate by tapping the protein using a cantilever (Ando et al. 2013). High-speed AFM provides real-time video of the protein in solution at nm-resolution on the second or sub-second timescale, and has been used to investigate the polymers of LC proteins (Babinchak et al. 2019) and gels of protein fibers (Cui et al. 2019; Wang et al. 2019). More recently, high-speed AFM has been utilized in the observation of the protein in the LLPS droplet (Fujioka et al. 2020).

For structure determination of polymers of LC proteins, exploitation of solid-state NMR (Murray et al. 2017; Murray and Tycko 2020), X-ray diffraction, and micro-electron diffraction (micro-ED) (Guenther et al. 2018; Luo et al. 2018) have been applied. Formation of the cross-β polymer was also observed for the FUS LC domain in the solid phase through solid-state NMR (Murray et al. 2017; Murray and Tycko 2020). In addition to single particle cryo-electron microscopy (cryo-EM) analysis, micro-ED is one of the recent emerging techniques in the field of structural biology (Nannenga and Gonen 2019). In micro-ED, the electron diffraction is collected for micro-3D protein crystals. Since only a-few-micrometer-size crystals give high-resolution electron density maps, micro-ED reduces the requirement crystal size for structure determination of proteins. We believe the next step is to try applying these electron-based imaging techniques in studies on proteins in LLPS droplets.

## Conclusion

In recent decades, there has been increased knowledge on the crucial role biological phase separation plays in bridging several gaps. It has addressed the gap between molecular biology at the genetic level, cell biology at the cellular level, and structural biology at the atomic level. While more and more biological processes can now be explained by LLPS phenomena, a large knowledge gap still remains with regard to understanding cellular LLPS. Further development of detection methods and novel approaches is required to better understand biological process effected and affected through biological phase separation.

## Appendix

**Table 1:** List of relevant abbreviations by section

The formation and regulation of membrane-less cellular organelles	pp. 2
Abbreviation	Full description
IDR	Intrinsically disordered region
LC domain	Low-complexity domain
LLPS	Liquid-liquid phase separation
P-body	Processing body
PML Body	Promyoelocytic leukemia body
rRNA	Ribosomal RNA
Stress granule assembly, regulation, and diseases	pp. 2–4
Abbreviation	Full description
ALS	Amyotrophic lateral sclerosis
C9orf72	Chromosome 9 open reading frame 72
cNLS	Canonical NLS
FG-repeats	Phenylalanine-Glycine repeats
FUS	Fused in sarcoma
G3BP1	G3BP stress granule assembly factor 1 or GTPase-activating protein (SH3 domain)-binding protein 1
GA	Glycine-Alanine
GP	Glycine-Proline
GR	Glycine-Arginine
hnRNPA1	Heterogeneous nuclear ribonucleoprotein A1
HRE	Hexanucleotide repeat expansion
HSP70	Heat shock protein 70
Impα/β	Importinα/β
Kapβ	Karyopherinβ
Kapβ2	Karyopherinβ2
MBP	Maltose-binding protein
NIR	Nuclear import receptor
NLS	Nuclear localization signal
PA	Proline-Alanine
PR	Proline-Arginine
PY-NLS	Proline-tyrosine NLS
RBP	RNA-binding protein
SG	Stress granule
TDP-43	Transactive response DNA-binding protein of 43 kDa
A new view of chromatin behavior	pp. 4–5
Abbreviation	Full description
BRD4	Bromodomain-containing protein 4
Modulating chromatin structure—compaction and decompaction	pp. 5
Abbreviation	Full description
C-terminal	Carboxy-terminal or COOH-terminal
CD	Chromodomain
CRISPR	Clustered regularly interspaced short palindromic repeats
CSD	Chromoshadow domain
H3K9me3	Trimethylation of histone H3 lysine 9
Hi-C	High-throughput chromosome conformation capture
hnRNPs	Heterogeneous nuclear ribonucleoproteins
HP1	Heterochromatin protein 1
SAF-A	Scaffold attachment factor A
Swi6	Chromoshadow domain of the *S. pombe* HP1 protein
Distinct microenvironment on chromatin	pp. 6
Abbreviation	Full description
CTD	C-terminal domain
EWS	Ewing sarcoma breakpoint region 1
FET family proteins	FUS, EWS, and TAF15
lncRNA	Long non-coding RNA
MED1	Mediator complex subunit 1
NEAT1	Nuclear Enriched Abundant Transcript 1
pol II	RNA polymerase II
TAF15	TATA-binding protein-associated factor 2 N
Tsix	The antisense repressor of XIST
XIST RNA	X-inactive specific transcript RNA
Aqueous two-phase systems	pp. 7–8
Abbreviation	Full description
ATPS	Aqueous two-phase system
PEG	poly (ethylene) glycol
Polyester microdroplets	pp. 8–9
Abbreviation	Full description
αHA	Alpha hydroxy acid
Techniques to observe structure and dynamics of phase-separated proteins	pp. 8–9
Abbreviation	Full description
FRAP	Fluorescence recovery after photobleaching
Solution nuclear magnetic resonance (NMR)	pp. 9–10
Abbreviation	Full description
HSQC	Heteronuclear single quantum coherence spectroscopy
NMR	Nuclear magnetic resonance
PCS	Pseudocontact shift
PFG-NMR	Pulsed-field gradient-nuclear magnetic resonance
PRE	Paramagnetic relaxation enhancement
RDC	Residual dipolar coupling
TROSY	Transverse relaxation optimized spectroscopy
Electron paramagnetic resonance (EPR)	pp. 10
Abbreviation	Full description
DEER	Double electron-electron resonance
EPR	Electron paramagnetic resonance
SDSL	Site-directed spin label
Other biochemical and biophysical methods	pp. 10–11
Abbreviation	Full description
AFM	Atomic force microscopy
Cryo-EM	Cryo-electron microscopy
Micro-ED	Micro-electron diffraction
NAI	N-acetylimidazole
SILAC	Stable isotope labeling by/with amino acids in cell culture
